# How the spleen reshapes and retains young and old red blood cells: A computational investigation

**DOI:** 10.1371/journal.pcbi.1009516

**Published:** 2021-11-01

**Authors:** He Li, Zixiang Leonardo Liu, Lu Lu, Pierre Buffet, George Em Karniadakis

**Affiliations:** 1 School of Engineering, Brown University, Providence, Rhode Island, United States of America; 2 Division of Applied Mathematics, Brown University, Providence, Rhode Island, United States of America; 3 Department of Chemical and Biomolecular Engineering, University of Pennsylvania, Philadelphia, Pennsylvania, United States of America; 4 Université de Paris, Inserm, Biologie Intégrée du Globule Rouge, Paris, France; University of California San Diego, UNITED STATES

## Abstract

The spleen, the largest secondary lymphoid organ in humans, not only fulfils a broad range of immune functions, but also plays an important role in red blood cell’s (RBC) life cycle. Although much progress has been made to elucidate the critical biological processes involved in the maturation of young RBCs (reticulocytes) as well as removal of senescent RBCs in the spleen, the underlying mechanisms driving these processes are still obscure. Herein, we perform a computational study to simulate the passage of RBCs through interendothelial slits (IES) in the spleen at different stages of their lifespan and investigate the role of the spleen in facilitating the maturation of reticulocytes and in clearing the senescent RBCs. Our simulations reveal that at the beginning of the RBC life cycle, intracellular non-deformable particles in reticulocytes can be biomechanically expelled from the cell upon passage through IES, an insightful explanation of why this peculiar “pitting” process is spleen-specific. Our results also show that immature RBCs shed surface area by releasing vesicles after crossing IES and progressively acquire the biconcave shape of mature RBCs. These findings likely explain why RBCs from splenectomized patients are significantly larger than those from nonsplenectomized subjects. Finally, we show that at the end of their life span, senescent RBCs are not only retained by IES due to reduced deformability but also become susceptible to mechanical lysis under shear stress. This finding supports the recent hypothesis that transformation into a hemolyzed ghost is a prerequisite for phagocytosis of senescent RBCs. Altogether, our computational investigation illustrates critical biological processes in the spleen that cannot be observed *in vivo* or *in vitro* and offer insights into the role of the spleen in the RBC physiology.

## Author summary

The spleen, the largest secondary lymphoid organ in humans, not only fulfils a broad range of immune functions, but also plays an important role in red blood cell (RBC) life cycle. In this study, we perform a computational study to simulate the passage of RBCs through interendothelial slits (IES) in the spleen at different stages of their lifespan, a critical biological process that cannot be observed in humans. Our simulation results illustrate a specific role of spleen in shaping young RBCs, which points to a probable missing step in current *in vitro* RBC culture protocols that fail to generate a majority of typical biconcave RBCs. Our results also reveal that intra-splenic mechanical constraints likely contribute to the final clearance and elimination of aged RBCs. Altogether, we demonstrate that our computational model can provide mechanistic rationales for experimental studies, offer insights into the role of the spleen in the RBC physiology and help the optimization of *in vitro* RBC culture techniques.

## Introduction

The spleen, the largest secondary lymphoid organ in the human immune system, works as a drainage network that prevents pathogenic microorganisms from remaining and multiplying in the bloodstream through innate phagocytosis or adaptive responses operated by lymphocytes and antibodies [1, 2]. In addition to its immune functions, the spleen also serves as a primary blood filter that can sequester 30–40% of the circulating platelet pool, regulate plasma volume, and remove senescent or pathologically altered erythrocytes (red blood cells, RBCs) from the circulation [3–5]. The splenic parenchyma is made of white pulp nodules and sheaths—that contain mainly T and B lymphocytes—interspersed into the red pulp, a spongy tissue that accounts for 75% of the splenic volume [1, 4, 6]. The red pulp comprises splenic sinusoids, which are blood vessels juxtaposed with the connective tissue of splenic cords. About 10–20% of blood entering the spleen is directed into the so-called open circulation, where RBCs navigate slowly and come in very close contact to abundant red pulp macrophages that can recognize surface alterations of RBCs through ‘ligand-receptor’ interactions and engulf the senescent and pathologically altered RBCs through phagocytosis [7–9]. In order to return to the general circulation, RBCs are forced to travel from the cords into venous sinuses, a process where RBCs have to squeeze through narrow apertures, interendothelial slits (IES), between elongated endothelial cells that form the sinus wall [10–12]. Since the splenic IES are narrower and shorter than capillaries, RBCs have to undergo severe deformation when traversing IES [13, 14]. As a result, RBCs with compromised deformability, such as the aged RBCs, are retained mechanically by IES. These sequential processes are a part of the spleen function to constantly control the quality of circulating RBCs.

The spleen, along with liver and bone marrow, is considered as the primary organs for clearing the senescent RBCs from circulation, although the underlying mechanism of the removal process is not fully understood [15]. During their ∼120 days’ lifespan in circulation, RBCs undergo progressive changes in cell morphology, membrane rigidity and expression of membrane proteins. The aged RBCs tend to become stiffer with cell shape gradually transforming from biconcave to spherical shape [16, 17]. Many membrane surface modulations are also observed on the aged RBCs, such as external exposure of membrane phosphatidylserine (PS) [18, 19], decreased levels of CD47 [20, 21], accumulation of anti-band 3 antibodies [22, 23]. These alterations have been regarded as senescence markers that are associated with the phagocytosis of aged RBCs by the splenic macrophages (erythrophagocytosis) [7]. Since the process of erythrophagocytosis cannot be observed *in vivo*, most of the hypotheses on the mechanism of RBC removal in the spleen are developed based on either *ex vivo* or *in vitro* studies where damaged or surface-altered RBCs are generated to mimic the aged RBCs. On one end, a number of *in vitro* [24–27] and *ex vivo* [28–30] experiments have demonstrated that RBCs with compromised deformability are mechanically retained at IES, susceptible to clearance by splenic macrophages. On the other end, *in vitro* investigations of RBC-macrophage interaction indicate that the biochemical markers on the membrane of RBCs, such as binding of antibodies (NAbs) to the band-3 proteins [22, 31], increased exposure of PS [32, 33], decreased expression of CD47 [20, 21] and conformational changes in CD47 [7, 20], can trigger the binding of senescent RBCs to macrophages and initiate the phagocytic processes. However, it is difficult to quantify the contribution of these morphological, biomechanical and biochemical markers to erythrophagocytosis as RBCs with removal signals are mostly phagocytosed in the spleen, leaving few of them in circulation for analysis. A recent *in vitro* study [34] presented new evidence of macrophages showing strong preference of recognizing and phagocytosing lysed RBC over intact ones, implying that hemolysis could be a potential key step in erythrophagocytosis. But the mechanism triggering the lysis of aged RBCs in the spleen is not clear.

In addition to filtering senescent RBCs, the spleen contributes to the function of facilitating the maturation of young RBCs (reticulocytes) [35]. Human RBCs are produced through erythropoiesis, where hematopoietic stem cells in the bone marrow develop into RBCs via a series of maturation stages [36]. In the late stage of erythropoiesis, normoblasts expel nucleus and form reticulocytes. Young reticulocytes are confined to the bone marrow for ∼24 hours before their egress to circulation to complete their maturation [37]. Prior studies suggested that the spleen can retain the circulating reticulocytes for 1–2 days, during which the reticulocytes shed unwanted membrane proteins and intracellular inclusions as the last step of their maturation [7, 38–40]. However, the detailed mechanism of how intracellular inclusions are removed from the spleen is still under debate. Crosby [41] proposed that reticulocytes expel their intracellular inclusions and non-essential membrane proteins when passing through IES where non-deformable parts were stuck and subsequently amputated from the cell, whereas De Back et al. deduced that the inclusions were cleared by splenic macrophages although the process of how macrophages eliminate the inclusions is elusive [7]. Moreover, clinical evidence suggests that the surface area of mature RBCs from splenectomized patients was significantly larger than that of nonsplenectomized normals, while the surface area difference between their reticulocytes was minimal [42]. This finding suggests that the spleen plays a role in reducing the redundant surface area of reticulocytes during their maturation, but how the surface area is removed from the reticulocytes in the spleen has not been addressed in detail. In the last decade, there has been an emerging prospect of using *in vitro* cultured, customizable RBCs for transfusion or drug delivery agents, but existing culture techniques are facing several challenges, such as the low yield of enucleated and biconcave RBCs [43]. The ability to promote the transformation from reticulocytes into more matured RBCs would likely optimize the survival and function of *in vitro* cultured RBCs after transfusion [44]. Thus, a better understanding of the processes involved in the final step of RBC maturation can provide new insights to improve *in vitro* culture systems.

In contrast to the extensive studies on the function of spleen in sensing and clearing diseased RBCs [25, 28, 29, 45–48], meager progress has been made in understanding the role of the spleen in erythropoiesis. In addition, prior studies on the traversal of RBCs through IES mainly look into the RBC deformation dynamics and the conditions for RBC retention or passage without considering the alterations of retained RBCs at IES.In this work, we perform a systematic computational study to simulate the passage of RBCs at different stages of their lifespan through IES in the spleen, the most stringent challenge on RBCs’ integrity and deformability in the human circulation. Different from prior computational work [14, 48–51], we primarily focus on simulating the alterations of reticulocytes and senescent RBCs when traversing IES, such as pitting, vesiculation and lysis, and explore the mechanism of the spleen in facilitating the maturation of reticulocytes as well as to examine the emerging hypothesis of intrasplenic hemolysis triggered by mechanical trapping of aged RBCs [34].

## Models and methods

**Fig 1:**
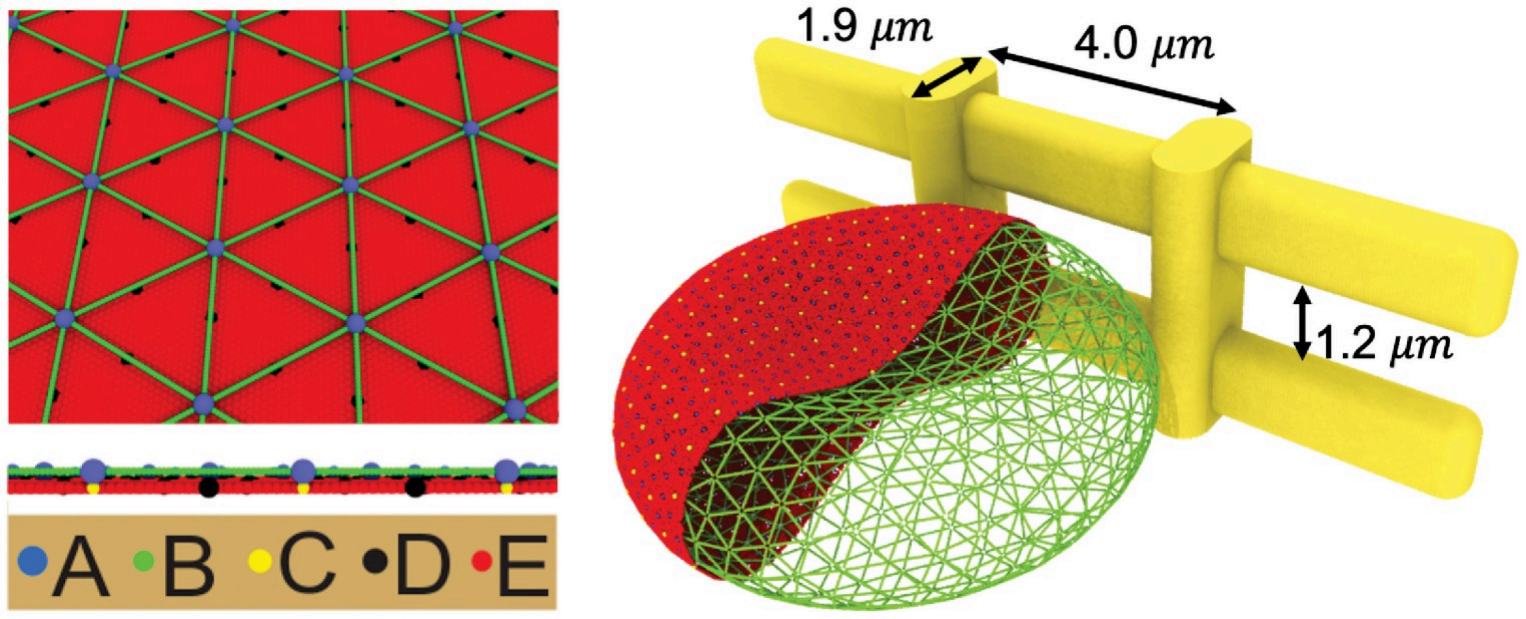
Simulating an RBC passing through IES by OpenRBC. The membrane of the RBC is explicitly represented by CG particles. A: actin junctions, B: spectrin particles, C: glycophorin particles, D: band-3 particles, E: lipid particles. The width and height of the simulated slit are 4.0 μm and 1.2 μm, respectively. The width of the vertical bars is 1 μm and the thickness of slit wall is 1.9 μm.

In the current work, we employ OpenRBC [52], a fast RBC simulator to simulate the reticulocytes, matured RBCs and aged RBCs. In OpenRBC, the lipid bilayer and cytoskeleton as well as the transmembrane proteins are explicitly represented. The cytoskeleton of the membrane, which consists of spectrin filaments connected at the actin junctional complexes forming a hexagonal network, as shown in Fig 1. The actin junctional complexes are represented by blue particles and they are connected to the lipid bilayer via glycophorin proteins. Spectrin is a protein tetramer formed by head-to-head association of two identical heterodimers. Each heterodimer consists of an *α*-chain with 22 triple-helical segments and a *β*-chain with 17 triple-helical segments and thus is represented by 39 spectrin particles connected by unbreakable springs. Three types of CG particles are introduced to represent the lipid bilayer of the RBC membrane. The red particles represent clusters of lipid molecules. The yellow particles underneath the blue particles represent glycophorin proteins and they are connected to the blue particles by unbreakable springs. The black particles signify band-3 proteins and they tether spectrin filaments to the lipid bilayer. This particle-based model has been widely used to study the biomechanics of RBC membrane under healthy and diseased conditions [53–57]. Different from RBC models that are constructed by one or two layers of 2D triangulated network [58–61], explicit representation of lipid bilayer and cytoskeleton by CG particles in the current model allows us to simulate the RBC lysis and membrane vesiculation in an explicit manner. More details about the RBC model can be found in S1 Text.

IES was built by using 4 solid bars, as illustrated in Fig 1. The two vertical bars represent annular fibers with widths of 1 *μ*m, whereas the two horizontal bars represent the elongated endothelial cells on the sinus wall. The thickness of the slit wall is 1.9 *μ*m. The width and height of the slit is 4.0 *μ*m and 1.2 *μ*m, respectively, which are consistent with the slit geometry employed in Pivkin et al. [48]. The boundaries of the bars are rounded with diameters equal to the thickness of the bar. We impose a repulsive interaction (L-J potential) at interfaces between the RBC and IES to prevent penetration of RBC particles into the slit wall.

## Results

### Mechanical interactions in the spleen can directly remove the inclusions from reticulocytes and contribute to their shape maturation

**Fig 2:**
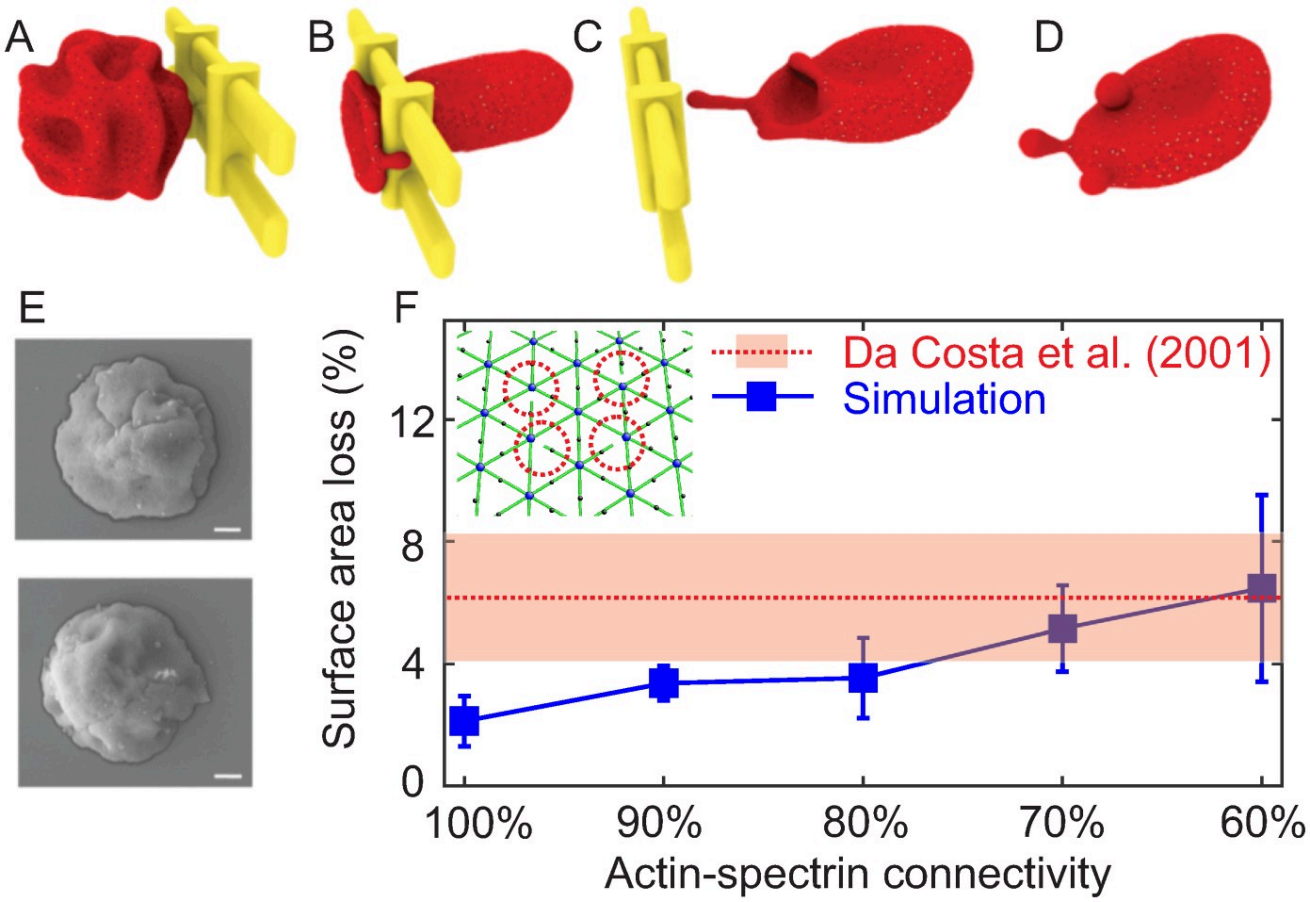
(A–D) Four sequential snapshots of a reticulocyte passing through IES driven by a pressure gradient of 10 Pa μm^−1^. The redundant membrane surface on the reticulocyte is removed through shedding vesicles. The reticulocyte develops to a biconcave shape after the passage of IES. (E) Two examples of cord blood reticulocytes with medium (top) and high (bottom) level of CD71 observed using scanning electron microscopy. Figure adopted from [65]. (F) Summary of the fraction of surface area loss from reticulocytes with various levels of actin-spectrin connectivity after passing through IES. The error bars are computed based on pressure gradient values of 5, 8, 10, 15 and 20 Pa μm^−1^. The red dashed line highlights the average fraction of reduced surface area of reticulocytes during their maturation as reported by Da Costa et al. [42]. The red area represents the standard deviation of the measurements.

In this section, we simulate the traversal of a reticulocyte through IES and investigate how IES in the spleen contributes to the maturation of reticulocytes. Guided by the experimental data reported in [62], we select the surface area and cell volume of the reticulocyte model to be 161.0 μm^2^ and 103.5 μm^3^, respectively. We also reduce the connections between the spectrin filaments and the actin junctions in the reticulocyte model (inset in Fig 2F) by 10%, 20%, 30% and 40%, respectively, to consider the effect of weaker association at the actin junction complexes in reticulocytes compared to the matured RBCs [63]. We have systematically studied the variations of the shear modulus and instability of reticulocyte model with respect to the reduced connectivity between the spectrin filaments and the actin junctions in our previous study [64], where we showed that the reduced connectivity leads to decreased shear modulus and increased instability of reticulocyte membrane (see S2 Text for more details). In this work, we focus on modeling the alteration of the reticulocytes induced by their traversal of IES. Following our prior work [47], we apply pressure gradients of 5, 8, 10, 15 and 20 Pa μm^−1^, respectively, to drive the reticulocytes through IES.

Fig 2A shows the equilibrium shape of a reticulocyte prior to its IES traversal; this reticulocyte model is characterized by deep membrane folds, consistent with experimental observations using scanning electron microscopy [65] (see Fig 2E), differential interference contrast microscopy [66] and phase contrast microscopy [67]. Fig 2B–2D illustrate typical deformation of a reticulocyte as it passes through IES. We note that the deep membrane folds on the reticulocyte disappear after it crosses IES (Fig 2D) and the reticulocyte develops into a biconcave shape. This reshaping process of the reticulocyte captured in our simulation demonstrates an example of how the spleen could play an important role in defining and determining the shape of RBCs, as previously hypothesized in Pivkin et al. [48]. Fig 2B–2D also show that the lipid bilayer detaches from the cytoskeleton when the reticulocyte traverses IES. Two detachments separate from the reticulocyte and form two vesicles, whereas the third one develops into a tubular vesicle and passes through IES following the reticulocyte. We note that these detachments are initiated at the locations where the actin-spectrin connection is disrupted (see S2 Fig). These results suggest that vesiculation is more likely to originate from the region where the lipid bilayer is not supported by the cytoskeleton and thus imply that shedding excessive surface area serves as a mechanism for optimizing the cohesion between lipid bilayer and cytoskeleton as reticulocytes mature [68]. This finding also shows consistency with the clinical evidence that the spleen may induce the removal of redundant membrane surface of reticulocytes to facilitate their maturation [42]. As shown in Fig 2E, when the actin-spectrin connectivity is reduced to 70% and 60%, the surface area loss from the traversing reticulocytes is comparable to the fraction of reduced surface area during the maturation of reticulocyte found experimentally [42]. These findings provide a plausible mechanism for the clinical evidence that RBCs from splenectomized patients were significantly larger than those of nonsplenectomized normals [42].

**Fig 3:**
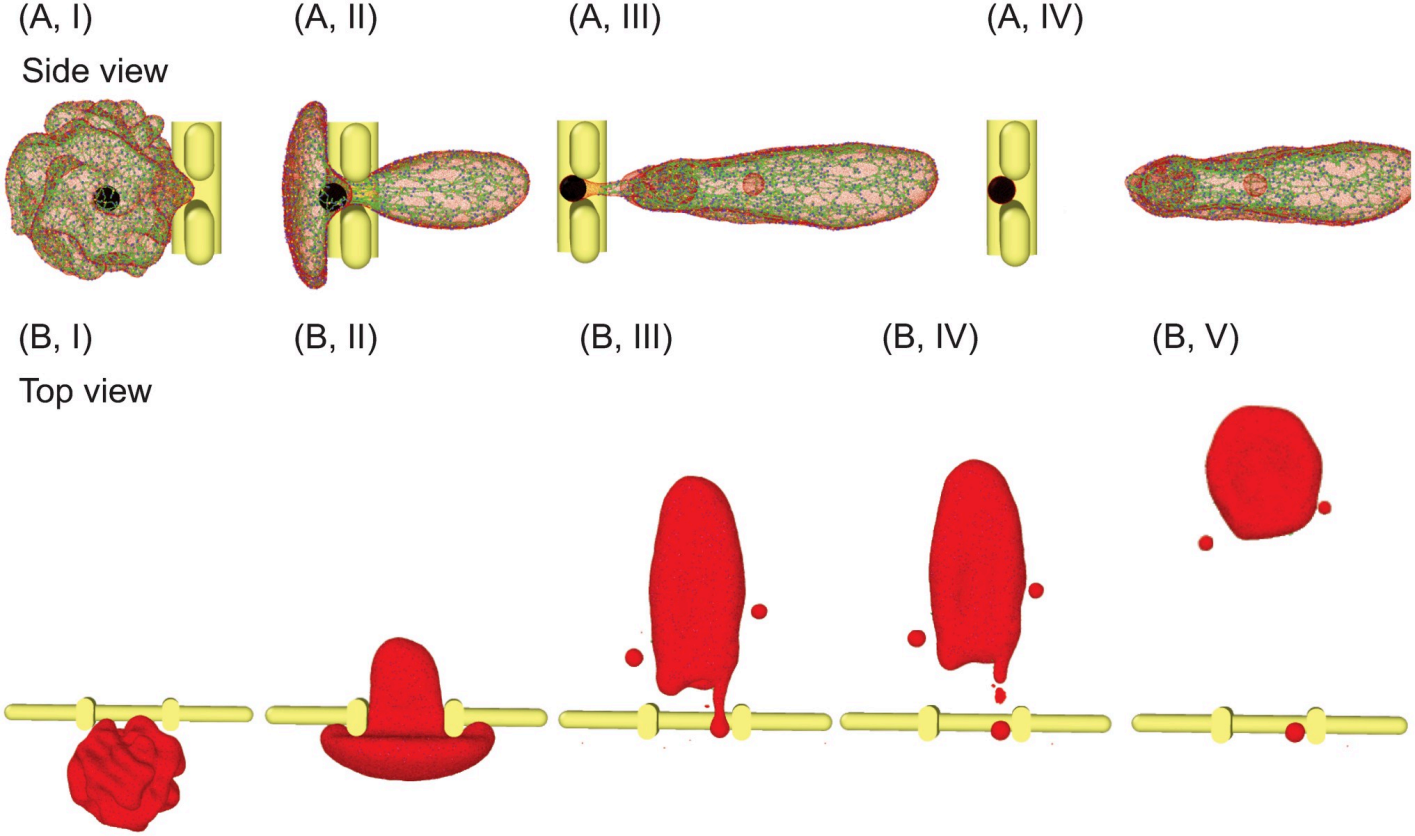
Sequential snapshots showing a spherical inclusion is removed from a reticulocyte during its passage through IES (A) side view and (B) top view. The reticulocyte is driven by a pressure gradient of 10 Pa μm^−1^. The spherical inclusion is simulated as a non-deformable particle with a diameter of 1.5 *μ*m. The lipid particles (red particles) in (A) are plotted at a smaller size to visualize the spherical inclusion (black particles).

Although the prevailing notion is that vestigial membrane proteins (e.g., CD71) and intracellular inclusions of reticulocytes are removed by red pulp macrophages in the spleen [7, 69], mechanical interaction between the reticulocytes and IES may also contribute to this removal process [41]. To this end, we simulate the passage of a reticulocyte containing an internal particle through IES to investigate the function of IES on removing inclusions. As shown in Fig 3A(I), we initially place a non-deformable spherical particle with a diameter of 1.5 *μ*m inside the reticulocyte model (black particle), mimicking an intracellular inclusion, such as a remnant of nuclei or a malaria parasite, with a size larger than the slit (1.25 *μ*m). We drive this reticulocyte through IES with a pressure gradient of 10 Pa μm^−1^. As illustrated in Fig 3A and 3B(I-V), the dynamics of this reticulocyte is noted with the disappearance of deep membrane folds, release of vesicles and shape transformation after crossing IES, similar to the ones without internal particles. Fig 3A(II) further shows that the internal particle is retained at IES while the reticulocyte is passing through. After the entire cell body crosses IES, the internal particle is wrapped only by the lipid bilayer (see Fig 3A(III)), forming a long tail on the moving cell (see Fig 3B(III)). As illustrated in Fig 3A(IV) and 3B(IV-V), the long tail eventually breaks up with the cell body, leaving the internal particle at IES. After traversing IES, the buds on the cell surface disappear and the elongated RBC transforms into biconcave shape. These simulation results confirm the hypothesis that the non-deformable inclusions in the reticulocytes can be stuck at IES in spleen and subsequently expelled from the cell by a biomechanical macrophage-independent process [41, 70].

### IES allows passage of normal RBCs

**Fig 4:**
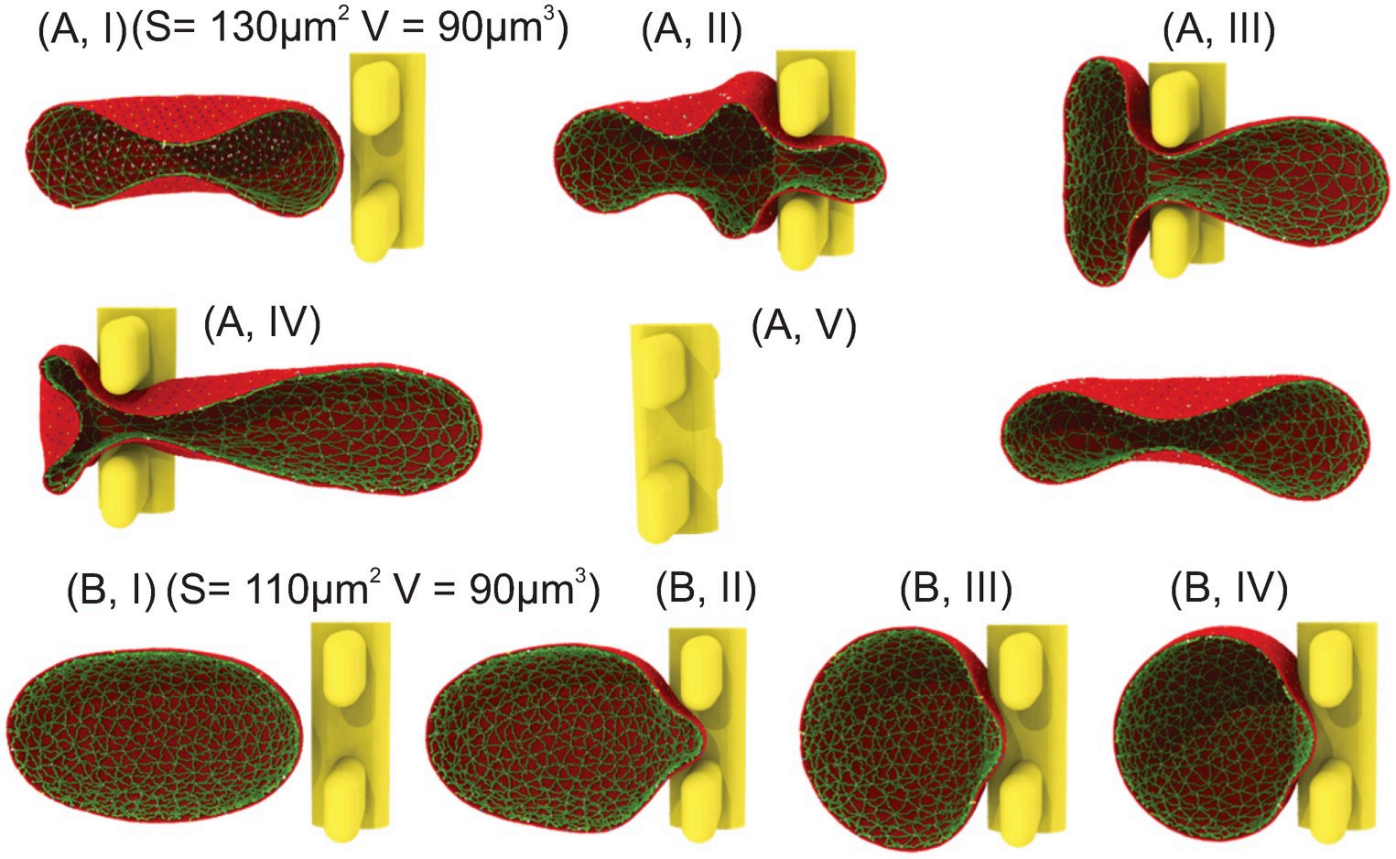
(A) Five successive snapshots of a normal RBC with surface area of 130 μm^2^ and cell volume of 90 μm^3^ passing through IES driven by a pressure gradient of 5 Pa μm^−1^. (B) Four successive snapshots of an RBC with surface area of 110 μm^2^ and cell volume of 90 μm^3^ moving toward IES and being retained at IES at a pressure gradient of 8 Pa μm^−1^. Only one half of the RBC is shown for visualization.

In this section, we simulate mature RBCs with different surface areas passing through IES. At each pressure gradient, we identify the critical RBC surface area-to-volume ratio (S/V), below which RBCs are retained by IES. First, the surface area of the traversing RBC is selected to be 130 μm^2^ and the volume is maintained at 90 μm^3^, giving a S/V of 1.45 which falls within the physiological range of S/V reported for normal RBCs [71, 72]. Similar to the cases of reticulocytes, we apply pressure gradients of 5, 8, 10, 15 and 20 Pa μm^−1^, respectively, to drive RBCs through IES. Our simulation results show that RBCs are able to pass through IES under these examined driving pressures. Fig 4A illustrate a sequence of typical shape deformations of an RBC during its passage of IES. When the RBC moves into the slit (see Fig 4A(II)), the portion inside the slit is being squeezed whereas the rest of the RBC membrane is expanded to accommodate the excluded volume by the narrow slit. The RBC starts to form a dumbbell shape with two bulges located on both upstream (left) and downstream (right) side of the slit; see Fig 4A(III). As the RBC moves further through, the right bulge expands while the left bulge shrinks. As the left bulge further shrinks to a certain extent, the cell membrane in the slit folds inward to the cell body and creates a concave region, forming a bullet-shape RBC, as shown in Fig 4A(IV). After crossing the slit, the deformed RBC gradually spreads out the infolded membrane and restores the biconcave shape (see Fig 4A(V)). The observations on the dynamics of RBCs passing through IES are consistent with the findings reported from former *in vivo* [13], *in vitro* [26] and computational studies [14, 48, 49]. We further examine the RBCs with surface area of 120 μm^2^ and volume of 90 μm^3^ and our simulation results show that these RBCs are also able to traverse IES under the examined driving pressures.

### IES retains senescent RBCs and makes them susceptible to lysis

Over the lifespan of ∼120 days, RBCs constantly lose surface area through releasing vesicles [19, 73]. Senescent RBCs are often display a reduced S/V, which tends to alter their equilibrium shape towards a spherical shape [74]. To assess how the reduced S/V in senescent RBCs would affect their traversal behavior at IES, we simulate the passage of RBCs through IES with surface area of 110 and 100 μm^2^, respectively, representing 15.3% and 23% surface area reduction from our normal RBC cases. The cell volume is still maintained at 90 μm^3^. Pressure gradients of 5, 8, 10, 15 and 20 Pa μm^−1^ are applied to drive the RBCs through IES, respectively. Fig 4B depict the RBC with a surface area of 110 μm^2^ and volume of 90 μm^3^ (S/V = 1.22), attempting to traverse IES driven by a pressure gradient of 8 Pa μm^−1^. Fig 4B(I) shows that in contrast to the biconcave shape of normal RBCs (see Fig 4A), the equilibrium shape of this RBC transforms to an ellipsoidal shape due to the reduced surface area. As a result of this shape alteration, Fig 4B(II-IV) show that this RBC is quickly stuck upstream of IES under a pressure gradient of 8 Pa μm^−1^. Our simulation results also show that RBCs with surface area of 100 μm^2^ (S/V = 1.11) is not able to traverse IES either. These results are generally in agreement with former *ex vivo* investigations showing that RBCs with more than 18% average surface area loss are mostly entrapped in the spleen [29].

**Fig 5:**
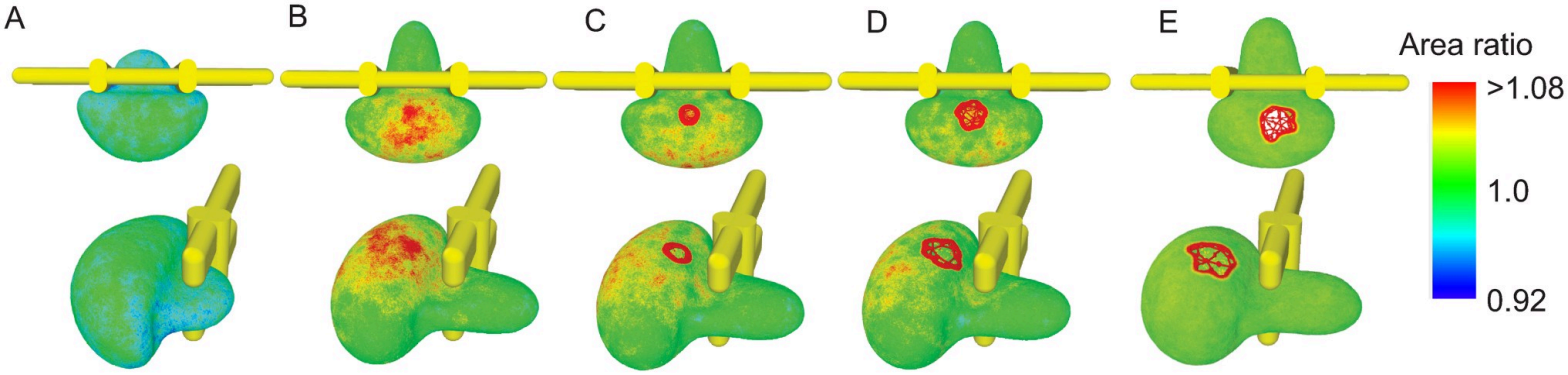
Five successive snapshots of an RBC with surface area of 110 μm^2^ and cell volume of 90 μm^3^ attempting to squeeze through IES at a pressure gradient of 10 Pa μm^−1^ (A) top view (B) side view. The RBC ruptures due to excessive local area expansion. The color contours show the local values of the area expansion ratio. Area ratio>1 indicates expansion whereas area ratio <1 indicates compression.

**Fig 6:**
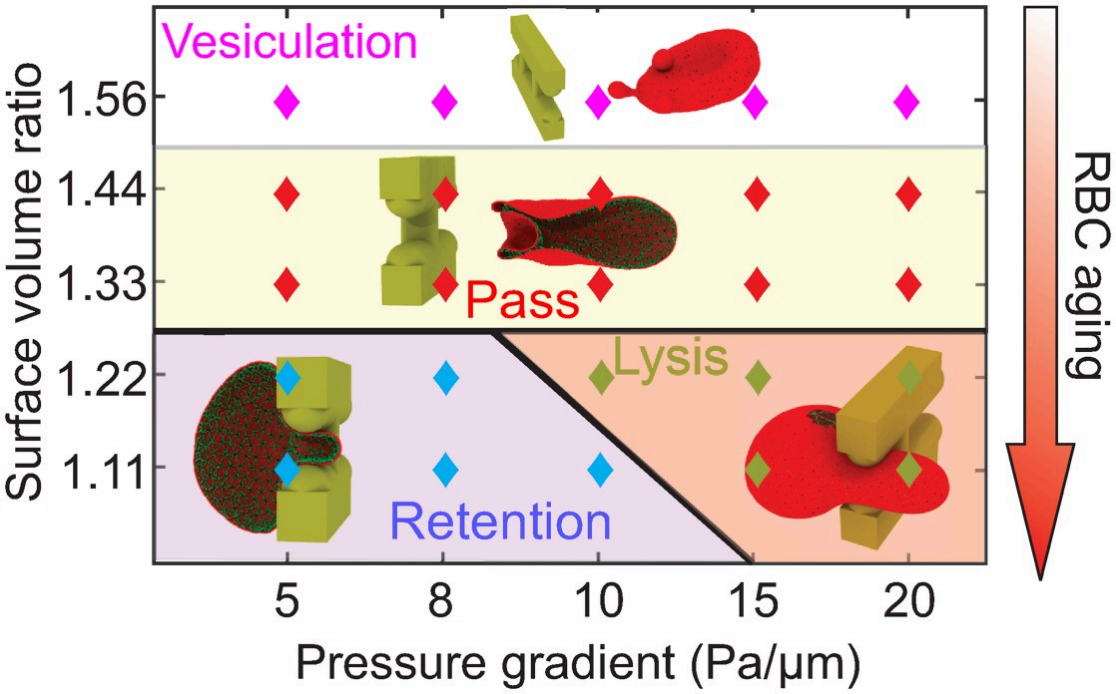
State diagram for RBC dynamics at IES driven by various pressure gradients and age-dependent RBC surface-to-volume ratios. Four states are observed. Red marks represent RBCs passing through IES. Pink marks represent the vesiculation of RBCs during their passage. Blue marks represent retention of RBCs by IES. Green marks represent lysis of RBCs at IES.

Next, we examine whether an increase in the local pressure gradient can force the retained RBCs pass through IES. Fig 5A–5E illustrate an RBC with a sub-normal surface area of 110 μm^2^ squeezing through the slit at an elevated pressure gradient of 10 Pa μm^−1^ from the case shown in Fig 4B. The color contours in the figures depict the local surface area expansion of the lipid bilayer. Fig 5B shows that area expansion occurs mostly on the big bulge that is upstream to IES. As the RBC moves through IES, the increasing local area expansion on the bulge leads to formation of a pore on the RBC lipid membrane at the site where the local area expansion peaks (Fig 5C). As the membrane is under significant expansion, the pore further expands and eventually causes the lysis of the RBC (see Fig 5D and 5E). Similar processes of pore formation on membrane patches under stretch were observed in [75–77]. Lysis also occurs for other retained RBCs with S/V = 1.11 and 1.22 when the driving pressure gradient is enhanced and the results are summarized in Fig 6. These results suggest that instead of forcing the retained senescent RBCs cross IES, the excessive pressure gradients cause RBC lysis due to the extreme local area expansion of lipid bilayer. This finding provides a plausible mechanism for the recent hypothesis that hemolysis is a key prerequisite for phagocytosis of senescent RBCs in the spleen [34]. We note that previous experimental studies reported that under quasi-static conditions, the membrane area of RBCs can expand by 2%–4%, beyond which the membrane may rupture [78, 79]. Under dynamic load, however, the critical value for the hemolysis could increase to as much as ∼40%, depending on the exposure time [80, 81]. According to the *in vivo* observations on the rate spleen by MacDonald et al. [13], the transit time of RBCs crossing a slit could range from 0.02 s to 60.5 s, suggesting that the critical area expansion value that triggers lysis in the spleen could vary widely for individual RBCs. Our simulations predict that the cell membrane ruptures when the local area expansion rate exceeds ∼8%, falling into the range of the critical area expansion for lysis reported in quasi-static and dynamic conditions in experimental [78–81] and computational [75] studies.

### Surface-to-volume ratio and local pressure gradient dictate the dynamics of RBCs at IES

Here, we summarize and map out the state diagram of the RBC dynamic behavior through IES as a function of the cell S/V (corresponding to the aging stages of RBCs) and pressure gradient across IES. As shown in Fig 6, our results show that S/V can dictate the passage of RBCs through IES. RBCs with S/V ratio being above a certain threshold (e.g., values between 1.22 and 1.33 as predicted in our simulations) could travel through IES while below this threshold value, RBCs may be retained or lysed depending on the local IES pressure gradient. Those RBCs, typically corresponding to senescent RBCs, are more likely to undergo erythrophagocytosis in the spleen. With S/V ranging between 1.25∼1.50, RBCs pass through IES with mainly reversible alteration of the RBC morphology and preserve the membrane integrity. Such healthy and mature RBCs tend to restore their original biconcave shape after returning to the post-sinus venules for normal functionality. For RBCs with excessive S/V, typically corresponding to reticulocytes, vesiculation of the RBC membrane could occur during their passage of IES. Such irreversible subtraction of the RBC membrane or inclusions contributes to the maturation or normalization of the RBC morphology. The observed state diagram, based on our computational results, points to the S/V ratio as one of the mechanics-based biomarkers adopted by the spleen to selectively sense and discharge healthy mature RBCs, while retaining or lysing the senescent RBCs.

## Discussion and summary

Our simulation illustrates that passage of IES allows reticulocytes to remove intracellular non-deformable particles, confirming a former hypothesis on the role of IES in removing the inclusions of reticulocytes [41, 70]. Our results also show that reticulocytes shed surface area through release of vesicles after crossing IES and transform into biconcave shape, an optimal morphology for the physiological function of RBCs in circulation. These results indicate that the spleen facilitates the removal of redundant surface area from the reticulocytes and thus expedites their maturation. This finding provides a rationale for the clinical evidence that RBCs from splenectomized patients is significantly larger than that of nonsplenectomized subjects [42]. It also points to the essential role of spleen in defining and determining the shape of RBCs [48]. Our simulation results also provide new insights to improve the current *in vitro* RBC culture protocols which are driven primarily by the intrinsic ability of erythroblasts to develop into reticulocytes. *In vitro* culture of fully enucleated, discocytic reticulocytes would likely optimize their survival and function in circulation [44].

The spleen is also one of the terminals for RBCs’ ∼120 day journey in circulation. As RBCs age, their deformability decreases primarily due to reduced S/V [82], increased membrane stiffness [83] and increased ratio of internal viscosity to external viscosity [84], see recent review in [85]. A number of experimental [29, 86] and computational studies [14, 48, 50, 51, 87] have demonstrated that S/V plays a much more important role in dictating the passage or retention of RBCs through narrow slit than the other two factors. In particular, a recent *ex vivo* experimental study [86] reported that a solo increase in membrane stiffness of diamide-treated RBCs without decreasing their S/V was not associated with mechanical retention in the human spleen. Subsequently, Lu and Peng [50] conducted a systematic computational study on the effects of reduced S/V, increased membrane stiffness and increased ratio of internal viscosity to external viscosity on the transit time of an RBC through a slit. Their results showed that while all the three factors can contribute to the prolonged transition time, an increase in S/V is more likely to cause RBC retention. The senescent RBCs are removed through erythrophagocytosis in the spleen, which are associated with not only biomechanical markers, but also biochemical markers [7, 69]. Prior *in vitro* experimental studies [8] suggested that the rigidity and shape of RBCs override the impact of CD47 in the process of phagocytosis, implying that less deformable RBCs are prioritized to undergo erythrophagocytosis in the spleen. A recent *in vitro* investigation provided new evidence showing a high propensity of macrophages on recognizing and phagocytosing lysed RBC over intact ones [34]. Our simulations results bridge the findings from these two separate experimental studies [8, 34] by showing the function of IES in retaining less deformable RBCs and making them susceptible to lysis. Our simulations also provide a mechanistic rationale for the hypothesis that hemolysis is a key event in the phagocytosis of senescent RBCs. Our findings along with previous *in vitro* studies support the mechanism that the senescent RBCs are first retained by IES and then undergo erythrophagocytosis to end their life cycle.

Although our simulation results demonstrate the function of the spleen on facilitating the maturation of reticulocytes, it does not exclude other mechanisms that also contribute to the final maturation process of reticulocytes in the spleen. For example, reticulocytes could expel unwanted membrane proteins such as transferrin receptor (CD71), CD98 and integrin *α*4*β*1, via releasing exosomes [88–91], which lead to decrease in cell surface area, volume and intracellular hemoglobin concentration [42, 92, 93]. We simulate the passage of an RBC containing an inclusion with a size of 1.5 *μ*m through IES (size of 1.2*μ*m) to examine a hypothesis raised by Crosby [41]. We note that not all inclusions in RBCs are as large as 1.5 *μ*m, but some are or come close to this size, such as remnants of nuclei (e.g., Howell-Jolly bodies). Any inclusions larger than the height of the slit, which could vary from 0.25 to 1.2*μ*m [10], may be removed by pitting [40, 94]. For example, large vacuoles observed by Differential Interference Contrast are removed by pitting [95]. Smaller inclusions could be removed when RBC cross narrower slits or through other mechanisms, such as interaction with splenic macrophages [7, 69]. In addition, prior *ex vivo* studies [5, 45] have shown that the malaria parasites invaded into the RBCs are retained by IES in the spleen and subsequently pitted from RBCs when squeezing through IES, a process that is captured by our computational model. These results demonstrate the biological and clinical relevance of our simulations. We note that the reticulocytes undergo drastic morphological changes during their maturation [67]. In the current study, we are attempting to simulate reticulocytes at their early stage of maturation, which are characterized by deep membrane folds, as observed in multiple experimental studies [65–67]. As reticulocytes mature, their degree of ‘foldness’ decreases through vesiculation either via traversing the spleen as demonstrated in the current work, or by undergoing high-shear flow in circulation until RBCs reach the optimal biconcave shape.

The pressure gradients we apply to drive RBCs through IES range from 5 to 20 Pa μm^−1^, which are greater than the critical pressure gradient of ∼1 Pa μm^−1^ found in microsphere experiments [24, 29]. This discrepancy could result from a difference between the size of the slits in the simulation and the size of the gaps between the microspheres in the experiment. In a separate microfluidic study, pressure gradients up to 30 Pa μm^−1^ were applied to drive RBCs through a slit with size down to 0.6 μm [26]. Although delicate *in vivo* measurements were performed by Atkinson and Sherlock [96] to assess the intrasplenic pressure, quantification of the pressure gradient across the sinus wall in the spleen still needs further investigation. In our simulation, we did not consider the dynamic remodeling of the RBC spectrin network [97, 98], because this process is regulated not only by biomechanical factors, but also by biochemical factors such as intracellular ATP (adenosine 5-triphosphate) and Ca^2+^ concentrations [99–101], which cannot be described by the current RBC model. As a result, no spectrin fragment is observed in the released vesicles. Metabolic remodeling of the RBC cytoskeleton could cause loss of cytoskeleton components into vesicles or the surrounding flow, as prior work reported that spectrin-free and band-3-rich vesicles are released from RBCs with ATP depletion [102] and Ca^2+^ loading [103], where the dynamic remodeling of cytoskeleton is suppressed. On the other hand, when subject to high temperature where the dynamic remodeling is encouraged, RBCs release vesicles containing cytoskeletal proteins such as spectrin and actin [104, 105]. We also note that we did not consider the impact of the drastic morphological alterations of RBCs due to either storage lesion [106, 107] or genetic mutations, such as echinocytes [108], sickle cells [109], acanthocytes and stomatocytes [110], on their passage of IES in the spleen, which can be targeted in future studies.

Taken together, we demonstrate that our computational model can simulate critical biological processes that cannot be observed *in vivo* or *in vitro* and offer insights into the role of the spleen in the RBC physiology. At the beginning of RBC’s life cycle, traversal of IES allows young RBCs to repel undesired inclusions, shed redundant surface area and transform to biconcave shape, facilitating their maturation. Towards the end of their life cycle, retention by IES makes the aged RBCs amenable to hemolysis, promoting their degradation. These findings can provide mechanistic rationales for experimental studies and guide the optimization of *in vitro* RBC culture techniques.

## Supporting information

S1 Fig Shear stress-strain responses of the reticulocyte membrane at decreased connectivity of the cytoskeleton. (TIF)

S2 Fig Six sequential snapshots of a reticulocyte passing through IES driven by a pressure gradient of 10 Pa μm^−1^. (TIF)

S1 Text Two-component RBC model. (PDF)

S2 Text Mechanical properties of reticulocyte model. (PDF)
