# Supplementary material for: How the spleen reshapes and retains young and old red blood cells: A computational investigation
Source: PLoS Comput Biol. 2021 Nov 1;17(11):e1009516. doi: 10.1371/journal.pcbi.1009516 (PMC8584971; doi:10.1371/journal.pcbi.1009516)
Supplement: S1 Text — (PDF) [file pcbi.1009516.s003.pdf]

S1_Text.Two-component RBC Model

In the coarse-grained molecular dynamics (CGMD) RBC model, the major two components of the RBC membrane, namely the cytoskeleton and the lipid bilayer, are represented explicitly by coarse grained (CG) particles. As shown in Fig 1 in the maintext, the cytoskeleton of the membrane consists of spectrin filaments connected at the actin junctional complexes, forming a hexagonal network. The actin junctional complexes, represented by blue particles, are con-nected to the lipid bilayer via glycophorin proteins (yellow particles). Spectrin is a protein tetramer formed by two identical heterodimers. Each heterodimer is comprised of an α-chain with 22 triple-helical segments and a β-chain with 17 triple-helical segments. Thus, each spec-trin filament is simulated by 39 spectrin particles (green particles). These spectrin particles are connected with unbreakable springs $u_{spectrin} = k_{spectrin}(d - d_{eq})^2$, where $k_{spectrin}$ is the spring constant, d and d_{eq} are distance and equilibrium distance between two spectrin CG particles. The lipid bilayer and transmembrane proteins of the RBC membrane are represented by three types of CG particles. The red CG particles denote aggregates of lipid molecules. The yellow particles signify glycophorin proteins which are connected to the blue particles by unbreakable springs. The black particles represent band-3 proteins that tether spectrin filaments to the lipid bilayer. These three types of CG particles interact via a pairwise potential similar to the Lennard-Jones potential. However, the employed interacting potential depends not only on the translational degrees of freedom of two interacting CG particles $\mathbf{d}_i$ and $\mathbf{d}_j$, but also on their rotational degrees of freedom $\mathbf{n}_i$ and $\mathbf{n}_j$. The potential is given by

$$u_{ij}(\mathbf{n}_i, \mathbf{n}_j, \mathbf{x}_{ij}) = u_R(d) + A(\alpha, a(\mathbf{n}_i, \mathbf{n}_j, \hat{\mathbf{x}}_{ij}))u_A(d), \tag{1}$$

$$u_R(d) = 1.4\epsilon(\frac{d_c - d}{d_c - d_{eq}})^8, \tag{2}$$

$$u_A(d) = -2.8\epsilon(\frac{d_c - d}{d_c - d_{eq}})^4, \tag{3}$$

$$A(\alpha, a(\mathbf{n}_i, \mathbf{n}_j, \hat{\mathbf{x}}_{ij})) = 1 + \alpha(a(\mathbf{n}_i, \mathbf{n}_j, \hat{\mathbf{x}}_{ij}) - 1), \tag{4}$$

$$a(\mathbf{n}_i, \mathbf{n}_j, \hat{\mathbf{x}}_{ij}) = (\mathbf{n}_i \times \hat{\mathbf{x}}_{ij}) \cdot (\mathbf{n}_j \times \hat{\mathbf{x}}_{ij}) = \mathbf{n}_i \cdot \mathbf{n}_j - (\mathbf{n}_i \cdot \hat{\mathbf{x}}_{ij})(\mathbf{n}_j \cdot \hat{\mathbf{x}}_{ij}), \tag{5}$$

where $\mathbf{x}_{ij} = \mathbf{d}_j - \mathbf{d}_i$, $d = |\mathbf{x}_{ij}|$ and $\hat{\mathbf{x}}_{ij} = \mathbf{x}_{ij}/d$. α is a parameter that tunes the bending stiffness of the RBC membrane. d_c is the cutoff distance of the potential and it is selected to be 2.6σ, where σ is the length unit of the system. ϵ is the energy unit. Actin and spectrin filaments interact with lipid bilayer and transmembrane proteins via a Lennard-Jones potential,

$$U_{LJ} = 4\epsilon\left[(\frac{\sigma}{d})^{12} - (\frac{\sigma}{d})^6\right] \quad d < d_{eq}. \tag{6}$$

Detailed information about this RBC model can be found in the authors' former work in Li et al. (1) and Tang et al. (2).

This RBC model can simulate an entire RBC by using ∼4 million CG particles using a single shared memory commodity workstation, but it is computationally expensive when simulating a long-time dynamic process such as a RBC passage through IES. In order to achieve higher computational efficiency, we apply a coarse model by using a fewer number of actin junctions in the RBC model. While the RBC membrane structure is preserved, we model 500 actin junctions in a single RBC , instead of a physiological value of ∼23867. Following the method applied in (3), the parameters in the coarse RBC model are recalibrated against experimental data to ensure the mechanical properties of the RBC are preserved. The coarse RBC model consists of 198965 CG particles and the length unit of the model is $\sigma = 35$ nm. The energy unit is $\epsilon = k_B T/0.22$, where k_B is the Boltzmann constant and the temperature of the system T is 300 K. The parameter that determines the bending stiffness of the RBC membrane, α, is selected to be 2.1. The translational motions of CG particles are governed by the Langevin equation

$$m_i \frac{d\mathbf{v}_i}{dt} = -\zeta \mathbf{v}_i + \mathbf{F}_i + \boldsymbol{\xi}_i, \tag{7}$$

where m_i and $\mathbf{v}_i$ are the mass and velocity of the CG particle i. $\mathbf{F}_i$ is deterministic force exerted on particle i and it results from the interacting potentials. ζ is the friction coefficient and it is selected to be $0.01m/\tau$, where τ is the time scale of the simulation and $m = 669$ kDa is the mass unit of the system. Selection of ζ will be further discussed in the following section. $\boldsymbol{\xi}_i$ is the random force, which has zero mean and variance of $2k_B T\zeta/\triangle t$. For numerical integration of the equations of motion, we use the velocity Verlet algorithm with a finite time step of $\triangle t = 0.01\tau$.

In this study, we simulate the young, mature and aged RBCs passage through IES using CGMD method where the timescale of the system cannot be calculated following the same strategy of molecular dynamics because CG particles represent a lump of atoms or molecules and thus are not real atoms. The correspondence between the simulation time and the physical time can be established via comparison with a referenced physical process. In our simulation, when driven by a pressure gradient of 5 Pa µm^{-1}, it takes approximately 190000τ for a RBC with surface area of 130 µm^2 and volume of 90 µm^3 to traverse IES. If we correspond this simulation time to the median transition time of 0.23 s for RBC passage through IES measured from *in vivo* studies of rate model (4; 5), τ can be calculated to be 1.21×10^{-6} s.

References

[1] Li H, Lykotrafitis G. Erythrocyte membrane model with explicit description of the lipid bilayer and the spectrin network. Biophysical Journal. 2014;107(3):642–653.

[2] Tang YH, Lu L, Li H, Evangelinos C, Grinberg L, Sachdeva V, et al. OpenRBC: A Fast Simulator of Red Blood Cells at Protein Resolution. Biophysical Journal. 2017;112(10):2030–2037.

[3] Pivkin IV, Karniadakis GE. Accurate coarse-grained modeling of red blood cells. Physical Review Letters. 2008;101(11):118105.

[4] Groom A, Schmidt E, MacDonald I. Microcirculatory pathways and blood flow in spleen: new insights from washout kinetics, corrosion casts, and quantitative intravital videomicroscopy. Scanning Microscopy. 1991;5(1):159–73.

[5] MacDonald IC, Ragan DM, Schmidt EE, Groom AC. Kinetics of red blood cell passage through interendothelial slits into venous sinuses in rat spleen, analyzed by *in vivo* microscopy. Microvascular Research. 1987;33(1):118–134.
