# Supplementary material for: How the spleen reshapes and retains young and old red blood cells: A computational investigation
Source: PLoS Comput Biol. 2021 Nov 1;17(11):e1009516. doi: 10.1371/journal.pcbi.1009516 (PMC8584971; doi:10.1371/journal.pcbi.1009516)
Supplement: S2 Text — (PDF) [file pcbi.1009516.s004.pdf]

S2_Text. Mechanical properties of reticulocyte model

The deformability and stability of reticulocytes increase as they mature (1; 2; 3). Maturing reticulocytes have been classified into different stages (4), which are featured with different morphologies and membrane shear moduli. Enucleation of late stage erythroblasts generates nascent reticulocytes, which are motile, multilobulated cells (R1 stage). As reticulocytes mature, they lose motility and transform to asymmetrical "deep dish" shaped cells with refractile ring and visible granules (R2 stage). During the subsequent maturation, the visible granules inside the reticulocytes disappear (R3 stage). Eventually, these "deep dish" shaped cells assume a biconcave shape. The shear modulus measured from the reticulocytes at different stages of maturation shows that the value of the shear modulus decreases from 11.4 μN/m (R1 stage) to 8.6 μN/m (R2 stage) and to 6.1 μN/m (R3 stage) (3). It is noted that the shear modulus of late stage reticulocytes (R3) is similar to the shear modulus of $3.7 \pm 2.3 \mu$N/m for mature RBCs (5). Although reticulocytes at R1 and R2 stages are stiffer than erythrocytes, they are less stable as reticulocytes break into fragments in the micropipette and ektacytometry experiments (1).

The shear moduli of the surface membrane of reticulocytes have been systematically studied in our previous study in (6). As displayed in S1 Fig , we show that the extended cytoskeleton in the reticulocyte membrane caused by excessive surface area of reticulocytes is responsible for its increased shear modulus. As the reticulocytes mature, the magnitude of its shear modulus reduces with the decreasing level of extension due to loss of surface area. We have validated these simulation results with data obtained from micropipette experiment performed in (5). In S1 Fig, we also show that the reduced actin-spectrin connectivity results in decreased reticulocyte rigidity. However, we note that the impact of extending the cytoskeleton on increasing the shear modulus in reticulocytes overwhelms the effect of compromised actin-spectrin connection on reducing the shear modulus, leading to more rigid reticulocyte membrane than the normal RBCs (see S1 Fig (D)). Similar observations are made in the malaria-infected RBCs where the adhesion of spectrin filaments to knobby area on the lipid membrane causes an enhanced stretch of cytoskeleton and thus results in an increased shear modulus although the actin-spectrin connectivity is reduced due to the loss of actin oligomers (7; 8).

References

[1] Chasis JA, Prenant M, Leung A, Mohandas N. Membrane assembly and remodeling during reticulocyte maturation. Blood. 1989;74(3):1112–1120.

[2] Waugh RE. Reticulocyte rigidity and passage through endothelial-like pores. Blood. 1991;78(11):3037–3042.

[3] Malleret B, Xu F, Mohandas N, Suwanarusk R, Chu C, Leite JA, et al. Significant biochemical, biophysical and metabolic diversity in circulating human cord blood reticulocytes. PLoS One. 2013;8(10):e76062.

[4] Mel HC, Prenant M, Mohandas N. Reticulocyte motility and form: studies on maturation and classification. Blood. 1977;49(6):1001–1009.

[5] Chu TT, Sinha A, Malleret B, Suwanarusk R, Park JE, Naidu R, et al. Quantitative mass spectrometry of human reticulocytes reveal proteome-wide modifications during maturation. British Journal of Haematology. 2018;180(1):118–133.

[6] Li H, Yang J, Chu TT, Naidu R, Lu L, Chandramohanadas R, et al. Cytoskeleton remodeling induces membrane stiffness and stability changes of maturing reticulocytes. Biophysical Journal. 2018;114(8):2014–2023.

[7] Zhang Y, Huang C, Kim S, Golkaram M, Dixon MW, Tilley L, et al. Multiple stiffening effects of nanoscale knobs on human red blood cells infected with Plasmodium falciparum malaria parasite. Proceedings of the National Academy of Sciences. 2015;112(19):6068–6073.

[8] Chang H, Li X, Li H, Karniadakis GE. MD/DPD multiscale framework for predicting morphology and stresses of red blood cells in health and disease. PLoS Computational Biology. 2016;12(10):e1005173.
